# Klippel–Feil Syndrome with Sprengel Deformity and Extensive Upper Extremity Deformity: A Case Report and Literature Review

**DOI:** 10.1155/2018/5796730

**Published:** 2018-01-18

**Authors:** John W. Stelzer, Miguel A. Flores, Waleed Mohammad, Nathan Esplin, Jonathan J. Mayl, Christopher Wasyliw

**Affiliations:** ^1^Department of Orthopaedic Surgery, Massachusetts General Hospital, Harvard Medical School, Boston, MA, USA; ^2^Department of Diagnostic Radiology, Florida Hospital, Orlando, FL, USA; ^3^University of Central Florida College of Medicine, Orlando, FL, USA

## Abstract

**Introduction:**

Klippel–Feil syndrome (KFS) is a congenital anomaly resulting from fusion of cervical vertebral bodies secondary to the dysregulation of signaling pathways during somite development. It is commonly associated with scoliosis and Sprengel deformity. We present a case of KFS with commonly associated abnormalities as well as deformities that have not yet been reported in the literature.

**Case Presentation:**

A 3-year-old girl presented for further evaluation of a left upper extremity deformity following a negative genetic workup. Upon physical exam and radiographic imaging, the patient was diagnosed with KFS and associated abnormalities including cervical scoliosis, Sprengel deformity, and congenital deformity of the left upper extremity. Deformities of the left upper extremity include radioulnar synostosis, a four-rayed hand, and absent thenar musculature. The Sprengel deformity was corrected surgically with a Woodward procedure.

**Discussion:**

Congenital musculoskeletal deformities can be differentiated based upon spinal and limb embryology. The presence of extraspinal abnormalities not originating from somite differentiation may suggest a severe form of KFS. Important considerations in the workup of the KFS patient include looking for deformities of the shoulder girdle and upper extremities to identify abnormalities for intervention at a young age.

## 1. Introduction

Klippel–Feil syndrome (KFS) is a congenital anomaly resulting from fusion of cervical vertebral bodies, characterized by the triad of cervical vertebral body fusion, low posterior hairline, and short neck with limited range of motion [1–3]. KFS is a rare condition, seen in approximately 1 in 40,000–42,000 live births with approximately equal distribution in males and females [4–6]. Pathogenesis of the disorder likely involves various dominant and recessive genetic mutations including *GDF6*, *GDF3*, *MEOX1*, and *RIPPLY2* which are responsible for transcription regulation and signaling pathways involved in somite development during embryogenesis [7–12]. KFS may be associated with other deformities, including Sprengel deformity (a congenitally high scapula), scoliosis, hearing impairment, congenital heart disease, lung defects, and genitourinary malformation [13, 14].

## 2. Case Presentation

The patient is a 3-year-old girl from China who initially presented with an ongoing diagnosis of left upper extremity deformity. Previous radiographs showed a deformity within the left forearm and hand, but left radial aplasia was excluded. Holt–Oram syndrome was previously excluded due to the lack of cardiac malformations and, more definitively, the lack of mutations within the *TBX5* gene. Klippel–Trenaunay syndrome was previously excluded due to the lack of a port-wine stain or other vascular malformations and the absence of limb or tissue overgrowth. Previous genetic testing revealed no mutations within the *PIK3CA* gene, making Klippel–Trenaunay syndrome unlikely.

Physical examination demonstrated a left hand with only four digits, likely from congenital fusion of the first and second digits which functioned as a thumb, opposing to the fifth digit with very good strength. The patient's left forearm measured 2 centimeters shorter than the right with apparent synostosis of the left proximal radioulnar joint. Additionally, the left humerus measured 3 centimeters shorter than the right. The patient was unable to undergo passive pronation or supination of the hand with preserved flexion and extension at the elbow joint. Examination of the patient's back demonstrated a symmetrically higher left scapula with a hard prominence palpable at the cervicothoracic junction.

**Figure 1:**
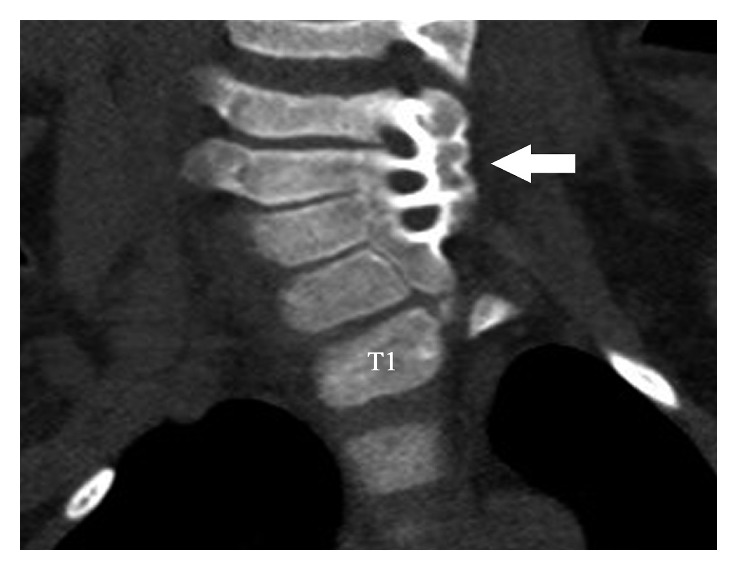
CT, coronal reformatted image demonstrates partial fusion involving the left cervicothoracic spine from C4 through T1 in a patient with Klippel–Feil syndrome (arrow). (*Courtesy of Miguel Flores*, *MD*, *Orlando*, *FL.*)

**Figure 2:**
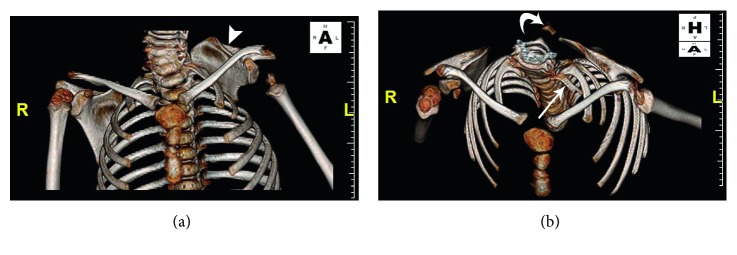
CT, 3D reconstructed images demonstrate Sprengel deformity in a patient with Klippel–Feil syndrome with abnormal elevation of the left scapula (a, arrowhead) and associated omovertebral bone (b, curved arrow). Incidental left cervical rib was also identified (b, arrow). (*Courtesy of Miguel Flores*, *MD*, *Orlando*, *FL.*)

**Figure 3:**
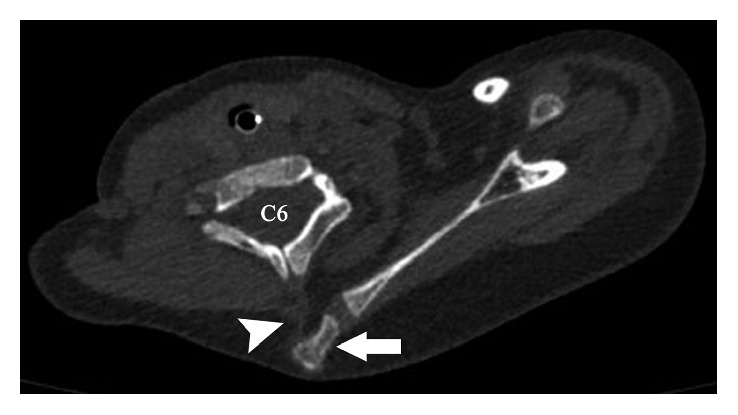
CT, axial image demonstrates Sprengel deformity with associated omovertebral bone (arrow) and fibrocartilaginous band (*a*rrowhead). (*Courtesy of Miguel Flores*, *MD*, *Orlando*, *FL.*)

**Figure 4:**
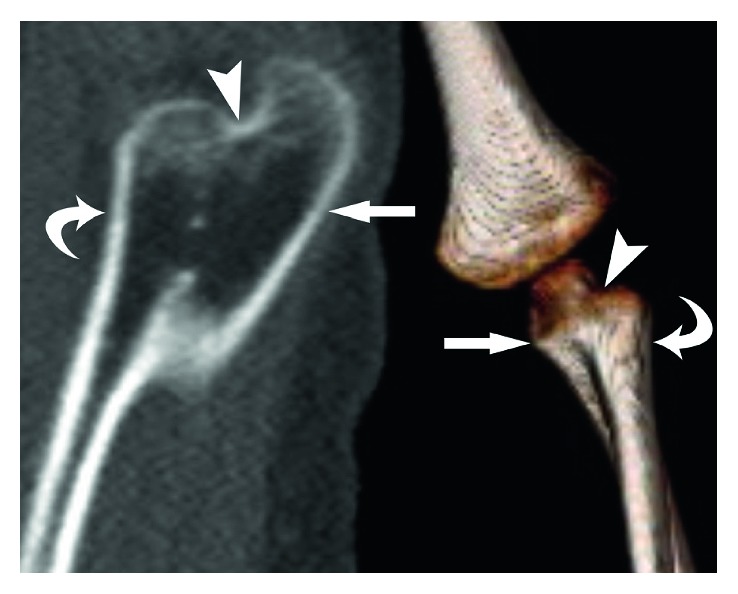
CT, sagittal reformatted (*left*) and 3D reconstructed (*right*) images demonstrate left radioulnar (radius = curved arrows, ulna = arrows) synostosis (arrowheads) in a patient with Klippel–Feil syndrome. (*Courtesy of Miguel Flores*, *MD*, *Orlando*, *FL.*)

Initial outside radiographs of the cervical, thoracic, and lumbar spine demonstrated mild scoliosis of the cervical spine. Further imaging revealed partial fusion of the left cervicothoracic spine from C4 to T1 (Figure 1). Elevation of the left scapula with an associated omovertebral bone was also noted (Figures 2 and 3). These findings are consistent with Klippel–Feil syndrome with an associated Sprengel deformity. Additional imaging of the left upper extremity confirmed a proximal radioulnar synostosis (Figure 4). Incidental findings included a left cervical rib and tracheal bronchus. The patient suffered no hearing impairment and no congenital cardiac or genitourinary defects upon further workup. Although genetic testing to further support a diagnosis of KFS was offered, the parents of the patient declined, since the immediate treatment plans would remain unchanged regardless of the results. Additional conditions considered in the patient's differential diagnosis included Poland syndrome and MURCS (müllerian duct aplasia-renal aplasia-cervicothoracic somite dysplasia) association; however, these were unlikely due to the lack of symptomology classically associated with the musculoskeletal deformities seen in each condition.

## 3. Discussion

Klippel–Feil syndrome was described over 100 years ago by Maurice Klippel and André Feil. However, opinions regarding associated abnormalities and treatment options are still evolving [2, 3]. The classic cervical vertebral abnormalities of KFS are well known and associated with derangements within the signaling pathways during paraxial mesoderm differentiation and somite development [12]. The literature also reports occurrences of KFS with common associated anomalies. Our case is unique due to the multiple extraspinal manifestations identified in a single patient, including Sprengel deformity and significant left upper extremity deformities such as proximal radioulnar synostosis and a four-rayed hand without thenar musculature. To the best of the author's knowledge, oligodactyly with absence of thenar musculature has not yet been reported with KFS.

Cervical scoliosis, which is the most common associated abnormality with KFS, was seen in the case presented. The patient also demonstrated partial fusion of the left cervicothoracic spine from C4 to T1 (Figure 1). Vertebral anomalies at the cervicothoracic junction are secondary only to the C2-C3 junction in prevalence of fusion anomalies [15]. The classification system recently proposed by Samartzis et al. defines the cervical spine fusion patterns for patients with KFS. The classification is determined radiographically such that Type I patients are defined as having a single congenitally fused cervical segment. Type II patients have multiple, noncontiguous congenitally fused segments, and Type III patients have multiple contiguous, congenitally fused cervical segments [16]. Under this proposed classification, our patient would be classified as a Type III KFS.

No surgical intervention, such as disc arthroplasty or fusion of unstable adjacent cervical spine levels, was indicated for our patient, since neurologic symptoms to suggest radiculopathy or myelopathy were not evident. However, Type III KFS patients do have increased risk of developing radiculopathic or myelopathic symptoms when compared to Type I and II patients [16]. Typical age of onset of spine-related neurologic symptoms is between 10 and 11 years of age for KFS patients when the disorder is identified in childhood. However, patients with milder forms of KFS not detected in childhood can present with neurologic symptoms into their 40s [16–18]. For this reason, the patient was encouraged to continue routine follow-up to evaluate for future development of neurological deficit.

In addition to cervical scoliosis, the presence of a Sprengel deformity was identified. This deformity, the second most common deformity associated with KFS, was first described by Eulenberg in 1863 [13, 19]. Years later, others described cases of the congenitally elevated scapula, but it was Otto Sprengel who described the pathology and proposed a theory of its existence in 1891 [20, 21]. The accepted cosmetic classification of Sprengel deformity, the Cavendish classification, was proposed in 1972 [22].

The Cavendish classification system proposed grades based on the deformity. Grade 1 is described as a very mild deformity that is not noticeable when the patient is dressed. Grade 2 is described as a mild deformity that is visible as a lump in the web of the neck when the patient is dressed. Grade 3 is a moderate deformity described as an easily visible deformity with the shoulder joint elevated 2–5 centimeters. Grade 4 is a severe deformity with shoulder joint elevation greater than 5 centimeters or evidence of the superior angle of the scapula near the occiput with or without webbing. Grading can be difficult because of the variation in appearance within a single grade. Although this classification does not consider function, it is utilized in the management of the deformity for objective differentiation when surgical intervention is necessitated to correct both appearance and function.

In the case of our patient, the left shoulder was elevated with scapular elevation to the level of C4-5 on CT imaging (Figure 2), translating clinically to a Grade 3 Sprengel deformity according to the Cavendish classification. The undescended scapula seen in Sprengel deformity is at times fixed in place to the adjacent vertebra by a pathognomonic omovertebral bone or fibrocartilaginous bridge preventing necessary scapular rotation during arm abduction past 90° (Figure 3) [23]. The arm is often unable to abduct and continue over the head due to the downward-facing glenoid cavity which may develop in the setting of a severely malrotated scapula.

The treatment for Sprengel deformity depends on the severity of the abnormality. For mild deformities classified as Cavendish Grades 1 and 2, nonsurgical options including physical therapy, stretching, and continued observation are most beneficial for the prevention of torticollis and decreased range of motion. Moderate and severe deformities that fall into the higher Cavendish classification grades are candidates for surgical intervention. Many surgical procedures for Sprengel deformity correction have been discussed in the literature, but the hallmark techniques involve resection of the omovertebral bone, if present, with caudal relocation of the scapula. Two of the most popular procedures are the Green's and Woodward procedures.

Green's procedure entails detaching muscles from their scapular insertion, elevating the trapezius muscle, and detaching the supraspinatus from the scapula followed by excision of the omovertebral bone. The supraspinous fossa of the scapula is resected, while being cautious not to injure the suprascapular neurovasculature, and the latissimus dorsi and serratus anterior are detached from the scapula as well. Once the scapula is descended to the corrected position, the muscles are reattached to it. Modifications have been made to the initial Green's procedure including a clavicular osteotomy to reduce the risk of brachial plexus injury, dissection of the insertion of the serratus anterior, and suturing of the inferior pole of the scapula to the thoracic cage into a pocket of the latissimus dorsi muscle [24].

The Woodward procedure was described in 1961 and is often the operation of choice for deformity correction. The procedure involves detaching the trapezius, rhomboid, and levator scapulae muscles at the midline origin followed by removing the omovertebral bone. Next, prominent bony portions of the scapula are removed as well, as the scapula is pulled downward and the muscle attachments are reattached distally to help secure the lowered scapula [25].

Surgical correction is recommended at a young age, usually between 3 and 8 years. However, a few studies have suggested that age does not influence outcomes [26, 27]. Since a higher-grade Sprengel deformity limits the patient's function by impeding necessary rotation of the scapula and shoulder girdle, surgical correction of the Sprengel deformity was indicated in our patient. Surgical correction would improve both function and aesthetics.

Limitations and complications specific to the surgical procedures for Sprengel deformity correction include hypertrophic scarring, regrowth of the resected bone, neurologic injury to the brachial plexus, and scapular winging [28–32]. Although cosmetic and functional improvements are not always optimally restored to normal, the improvements seen in the aesthetics and function of the scapula can be very significant. The mean arm abduction improvements in studies with correctional surgery for Sprengel deformity have been reported between 49° and 77°. Additionally, the mean improvement of Cavendish grading has been reported from 1.5 to 2.0 grades lower in follow-up studies after surgical correction [28, 33–38].

The Sprengel deformity was not the only musculoskeletal abnormality resulting in physical limitation. The patient's left upper extremity syndactyly and proximal radioulnar synostosis (Figure 4) only allowed for flexion and extension at the elbow joint. Pronation and supination were not possible secondary to the proximal radioulnar synostosis that kept the left arm fixed in 10 degrees of pronation. Surgical correction to restore pronation and supination, however, was not advised. Surgical correction for congenital radioulnar synostosis is rarely indicated except in cases of severe deformity (i.e., ≥60° of pronation) due to high recurrence rates and therefore was not performed [39–41].

## 4. Conclusion

Congenital musculoskeletal deformities can be differentiated based on mechanisms of spinal and limb embryology. The presence of extraspinal manifestations, not originating from somite differentiation, may be indicative of a more severe form of Klippel–Feil syndrome. Important considerations in the workup of the KFS patient include looking for deformities of the shoulder girdle and upper extremities. Identifying these associated abnormalities early is paramount to assess for potential surgical intervention at a young age.
